# Supplementary material for: Meta-analysis reveals that pollinator functional diversity and abundance enhance crop pollination and yield
Source: Nat Commun. 2019 Apr 1;10:1481. doi: 10.1038/s41467-019-09393-6 (PMC6443707; doi:10.1038/s41467-019-09393-6)
Supplement: Supplementary file 1 — Supplementary Information [file 41467_2019_9393_MOESM1_ESM.docx]

Supplementary information

Meta-analysis reveals that pollinator functional diversity and abundance enhance crop pollination and yield’ by Woodcock et al.

Woodcock et al.

Supplementary Figures 1: Correlations between metrics of pollinator community structure and oilseed rape yield.

Supplementary Methods: Supplementary methods cover: 1) Meta-analysis diagnostics; 2) Pearson’s correlations between effect traits and the mean pollen stigmal deposition; 3) PRISMA flow diagram showing the process of study selection for the meta-analysis; 4) Summary methodologies of unpublished studies describing the sampling of pollinator communities and the assessment of oilseed rape yield in response to pollination; 5) Definition of effect groups.

Supplementary Figures.

|  | **M1:** Jauker and Wolters ^1^ | **M2:** Jauker, et al. ^2^ | **M3:** Steffan-Dewenter ^3^ |
| --- | --- | --- | --- |
| **Yield (Mean=0, SD=1)** | 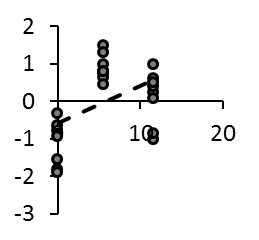 | 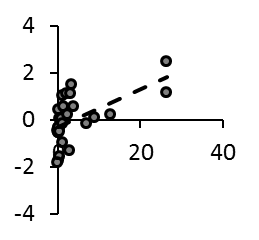 | 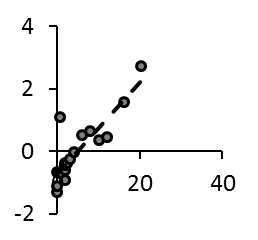 |
|  |  |  |  |
|  | **M4:** Steffan-Dewenter ^3^ | **M5:** Garratt, et al. ^4^ | **M6:** Soroka, et al. ^5^ - 1994 experiment |
|  | 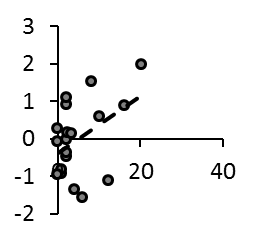 | **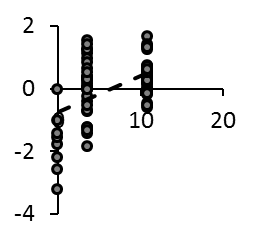** | 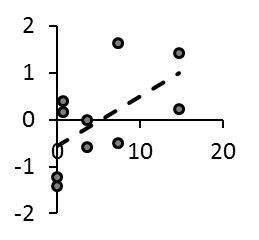 |
|  | **M7:** Soroka, et al. ^5^ - 1995 experiment |  |  |
|  | 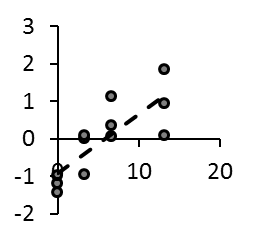 |  |  |
|  | **Abundance (corrected to one SD)** | | |

| **Yield (Mean=0, SD=1)** | **F1:** Lindström, et al. ^6^ | **F2:** Lindström, et al. ^6^ | **F3:** Bommarco, et al. ^7^ |
| --- | --- | --- | --- |
|  | 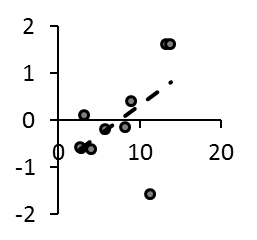 | 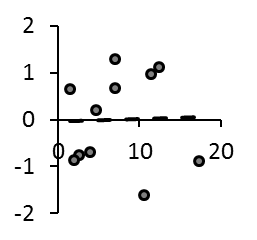 | 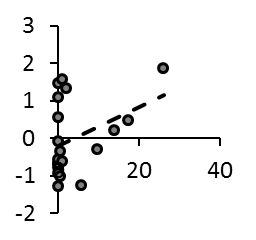 |
|  | **F4:** Wessex - 2013* | **F5:** Wessex - 2013* | **F6:** Hillesden - 2014* |
|  | 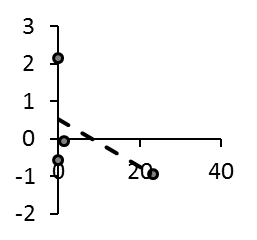 | 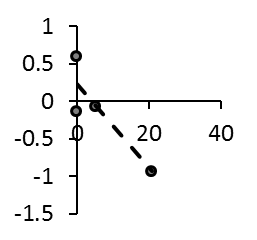 | 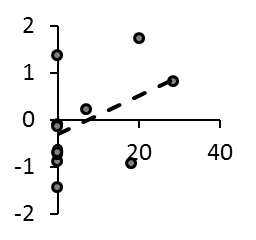 |
|  | **F7:** Salisbury - 2012* | **F8:** Woodcock, et al. ^8^ | **F9:** Woodcock, et al. ^8^ |
|  | 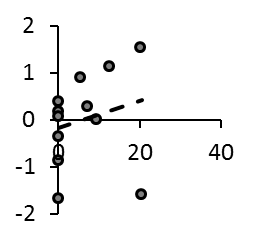 | 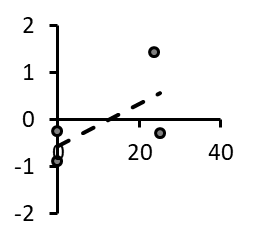 | 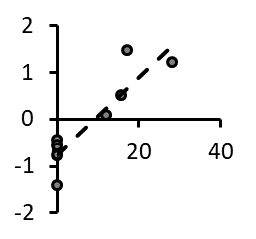 |
|  | **F10:** Waddesdon - 2013* | **F11:** Stanley, et al. ^9^ | **F12:** Morandin and Winston ^10^ |
|  | 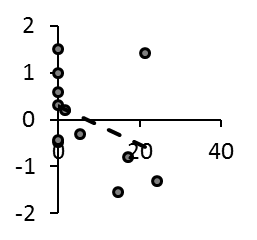 | 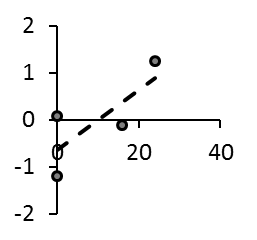 | 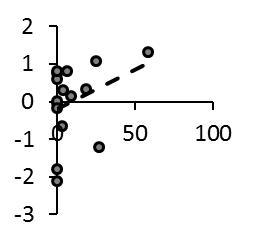 |
|  | **Abundance (corrected to one SD)** | | |

| **Yield (Mean=0, SD=1)** | **F13:** Morandin and Winston ^10^ | **F14:** Morandin and Winston ^10^ 2002 expt. | **F15:** Morandin and Winston ^10^ 2003 expt. |
| --- | --- | --- | --- |
|  | 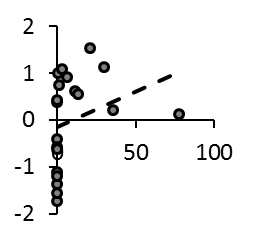 | 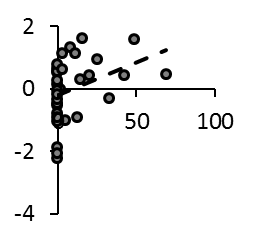 | 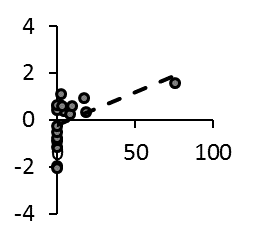 |
|  | **F16:** Zou, et al. ^11^ |  |  |
|  | 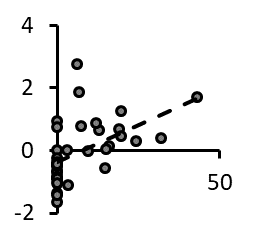 |  |  |
|  | **Abundance (corrected to one SD)** | | |

**Supplementary Figure 1a. Individual study abundance – yield scatter plots.** Scatter plots showing raw data relationship between yield (corrected SD=1) and pollinator community abundance for individual studies. Sub-panels refer to the individual studies of M1: Jauker and Wolters ^1^; M2: Jauker, et al. 2; M3: Steffan-Dewenter Express MSL ^3^; M4: Steffan-Dewenter Express ^3^; M5: Garratt, et al. ^4^; M6: Soroka, et al. ^5^ - 1994 experiment; M7: Soroka, et al. ^5^ - 1995 experiment; F1: Lindström, et al. ^6^; F2: Lindström, et al. ^6^; F3: Bommarco, et al. ^7^; F4: Wessex – DK Cabernet; F5: Wessex – PR46W21; F6: Hillesden – 2014 Excalibur; F7: Salisbury – 2012 DK Cabernet; F8: Woodcock, et al. ^8^ NK Molten; F9: Woodcock, et al. ^8^ DK Cabernet; F10: Waddesdon – 2013 Dimension; F11: Stanley, et al. ^9^; F12: Morandin and Winston ^10^ cv45A71; F13: Morandin and Winston ^10^ cvCL289; F14: Morandin and Winston ^10^ 2002 expt. cvDK3235; F15: Morandin and Winston ^10^ 2003 expt. cvDK3235; F16: Zou, et al. ^11^.

|  | **M1:** Jauker and Wolters ^1^ | **M2:** Jauker, et al. ^2^ | **M3:** Steffan-Dewenter ^3^ |
| --- | --- | --- | --- |
| **Yield (Mean=0, SD=1)** | 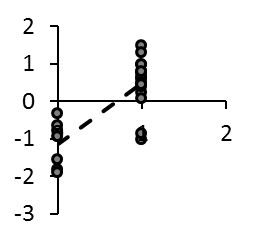 | 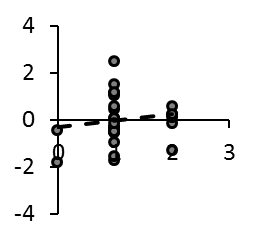 | 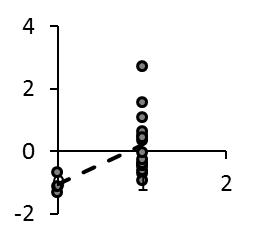 |
|  |  |  |  |
|  | **M4:** Steffan-Dewenter ^3^ | **M5:** Garratt, et al. ^4^ | **M6:** Soroka, et al. ^5^ - 1994 experiment |
|  | 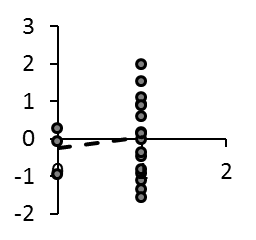 | **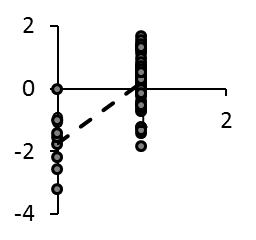** | 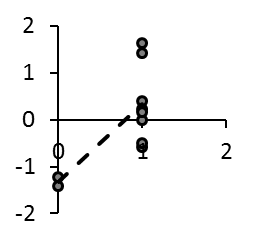 |
|  | **M7:** Soroka, et al. ^5^ - 1995 experiment |  |  |
|  | 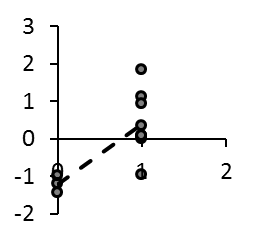 |  |  |
|  | **Species richness** | | |

| **Yield(mean=0, SD=1)** | **F1:** Lindström, et al. ^6^ | **F2:** Lindström, et al. ^6^ | **F3:** Bommarco, et al. ^7^ |
| --- | --- | --- | --- |
|  | 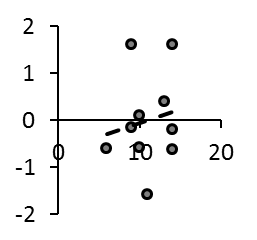 | 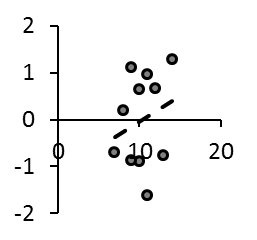 | 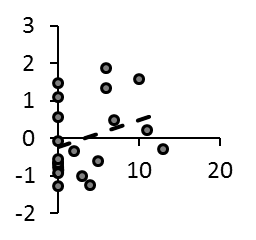 |
|  | **F4:** Wessex - 2013* | **F5:** Wessex - 2013* | **F6:** Hillesden - 2014* |
|  | 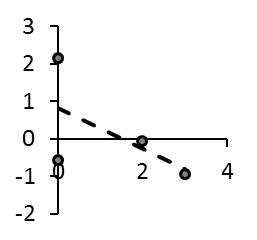 | 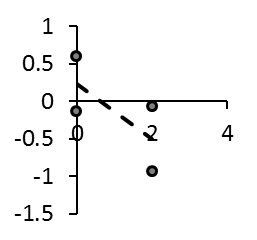 | 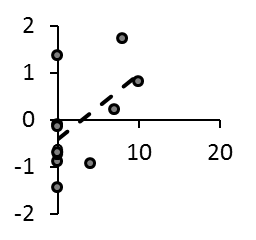 |
|  | **F7:** Salisbury - 2012* | **F8:** Woodcock, et al. ^8^ | **F9:** Woodcock, et al. ^8^ |
|  | 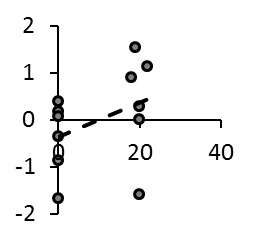 | 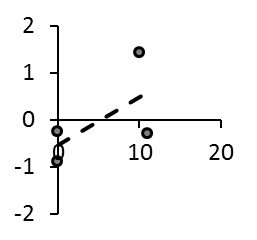 | 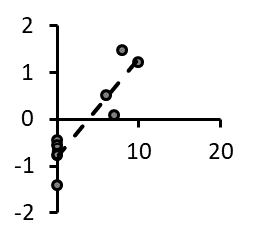 |
|  | **F10:** Waddesdon - 2013* | **F11:** Stanley, et al. ^9^ | **F12:** Morandin and Winston ^10^ |
|  | 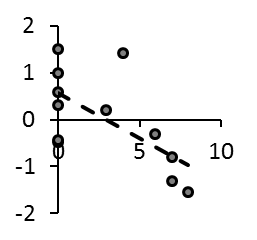 | 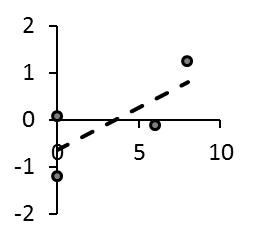 | 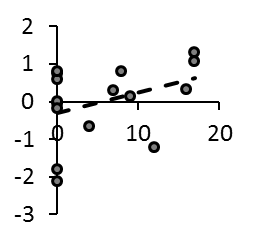 |
|  | **Species richness** | | |

| **Yield (Mean=0, SD=1)** | **F13:** Morandin and Winston ^10^ | **F14:** Morandin and Winston ^10^ 2002 expt. | **F15:** Morandin and Winston ^10^ 2003 expt. |
| --- | --- | --- | --- |
|  | 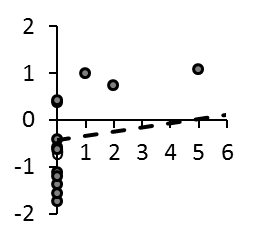 | 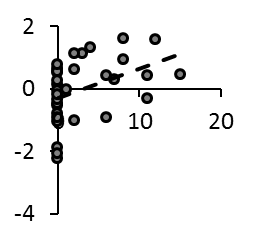 | 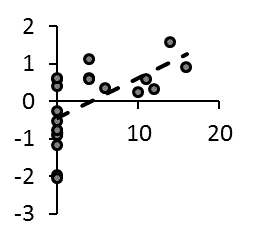 |
|  | **F16:** Zou, et al. ^11^ |  |  |
|  | 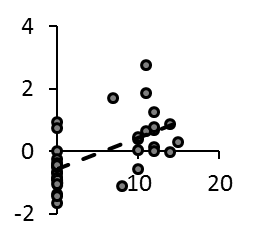 |  |  |
|  | **Species richness** | | |

**Supplementary Figure 1b. Individual study species rihcness – yield scatter plots.** Scatter plots showing raw data relationship between yield (corrected SD=1) and pollinator community species richness for individual studies. Sub-panels refer to the individual studies of M1: Jauker and Wolters ^1^; M2: Jauker, et al. 2; M3: Steffan-Dewenter Express MSL ^3^; M4: Steffan-Dewenter Express ^3^; M5: Garratt, et al. ^4^; M6: Soroka, et al. ^5^ - 1994 experiment; M7: Soroka, et al. ^5^ - 1995 experiment; F1: Lindström, et al. ^6^; F2: Lindström, et al. ^6^; F3: Bommarco, et al. ^7^; F4: Wessex – DK Cabernet; F5: Wessex – PR46W21; F6: Hillesden – 2014 Excalibur; F7: Salisbury – 2012 DK Cabernet; F8: Woodcock, et al. ^8^ NK Molten; F9: Woodcock, et al. ^8^ DK Cabernet; F10: Waddesdon – 2013 Dimension; F11: Stanley, et al. ^9^; F12: Morandin and Winston ^10^ cv45A71; F13: Morandin and Winston ^10^ cvCL289; F14: Morandin and Winston ^10^ 2002 expt. cvDK3235; F15: Morandin and Winston ^10^ 2003 expt. cvDK3235; F16: Zou, et al. ^11^.

| **Yield (Mean=0, SD=1)** | **F1:** Lindström, et al. ^6^ | **F2:** Lindström, et al. ^6^ | **F3:** Bommarco, et al. ^7^ |
| --- | --- | --- | --- |
|  | 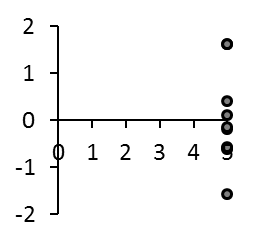 | 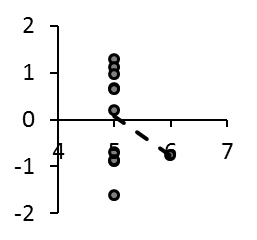 | 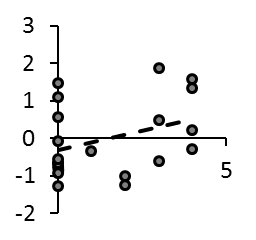 |
|  | **F4:** Wessex - 2013* | **F5:** Wessex - 2013* | **F6:** Hillesden - 2014* |
|  | 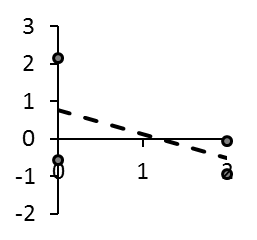 | 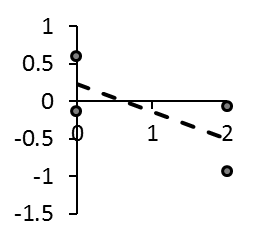 | 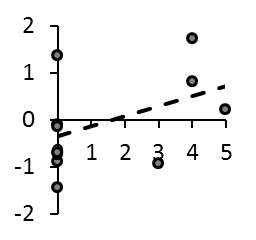 |
|  | **F7:** Salisbury - 2012* | **F8:** Woodcock, et al. ^8^ | **F9:** Woodcock, et al. ^8^ |
|  | 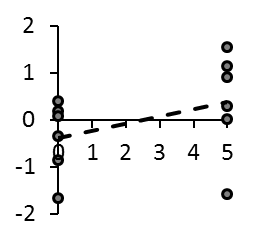 | 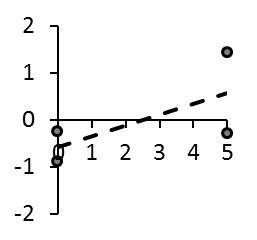 | 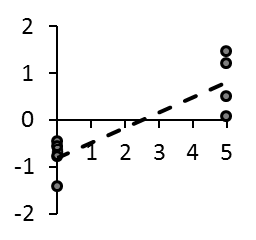 |
|  | **F10:** Waddesdon - 2013* | **F11:** Stanley, et al. ^9^ | **F12:** Morandin and Winston ^10^ |
|  | 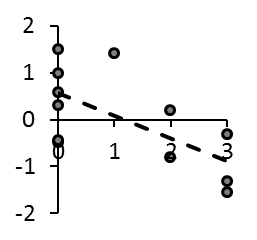 | 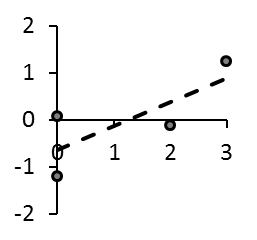 | 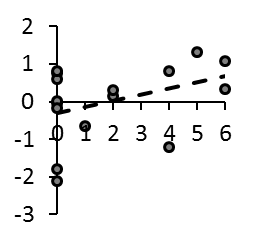 |
|  | **Effect group richness** | | |

| **Yield (Mean=0, SD=1)** | **F13:** Morandin and Winston ^10^ | **F14:** Morandin and Winston ^10^ 2002 expt. | **F15:** Morandin and Winston ^10^ 2003 expt. |
| --- | --- | --- | --- |
|  | 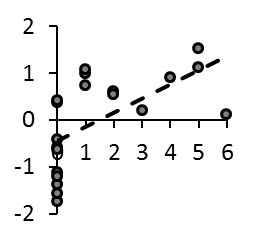 | 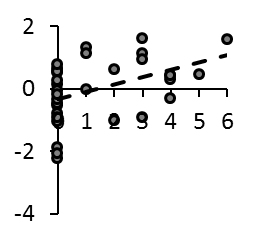 | 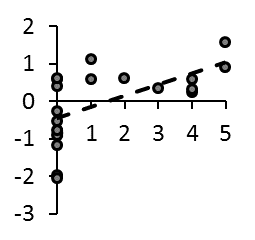 |
|  | **F16:** Zou, et al. ^11^ |  |  |
|  | 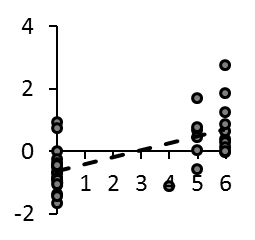 |  |  |
|  | **Effect group richness** | | |

**Supplementary Figure 1c. Individual study effect group richness – yield scatter plots.** Scatter plots showing raw data relationship between yield (corrected SD=1) and pollinator community effect group richness for individual studies. Sub-panels refer to the individual studies of F1: Lindström, et al. ^6^; F2: Lindström, et al. ^6^; F3: Bommarco, et al. ^7^; F4: Wessex – DK Cabernet; F5: Wessex – PR46W21; F6: Hillesden – 2014 Excalibur; F7: Salisbury – 2012 DK Cabernet; F8: Woodcock, et al. ^8^ NK Molten; F9: Woodcock, et al. ^8^ DK Cabernet; F10: Waddesdon – 2013 Dimension; F11: Stanley, et al. ^9^; F12: Morandin and Winston ^10^ cv45A71; F13: Morandin and Winston ^10^ cvCL289; F14: Morandin and Winston ^10^ 2002 expt. cvDK3235; F15: Morandin and Winston ^10^ 2003 expt. cvDK3235; F16: Zou, et al. ^11^.

| **Yield (Mean=0, SD=1)** | **F1:** Lindström, et al. ^6^ | **F2:** Lindström, et al. ^6^ | **F3:** Bommarco, et al. ^7^ |
| --- | --- | --- | --- |
|  | 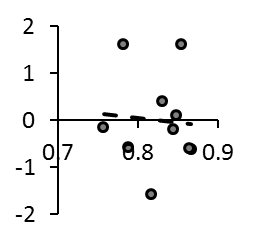 | 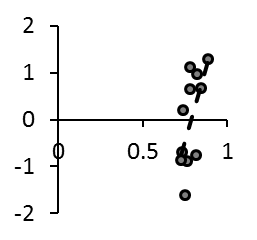 | 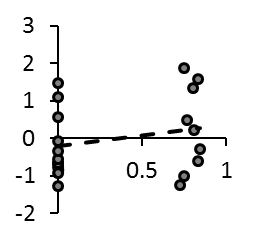 |
|  | **F4:** Wessex - 2013* | **F5:** Wessex - 2013* | **F6:** Hillesden - 2014* |
|  | 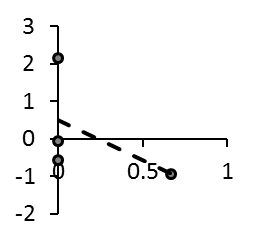 | 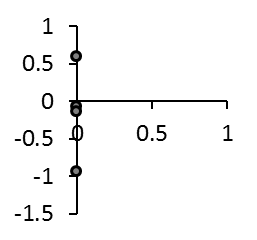 | 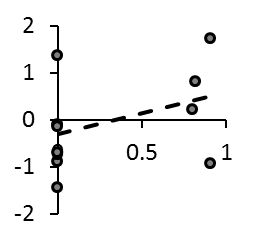 |
|  | **F7:** Salisbury - 2012* | **F8:** Woodcock, et al. ^8^ | **F9:** Woodcock, et al. ^8^ |
|  | 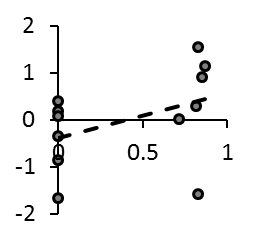 | 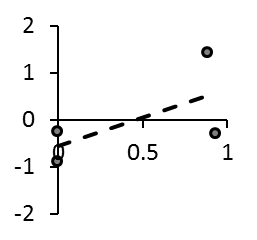 | 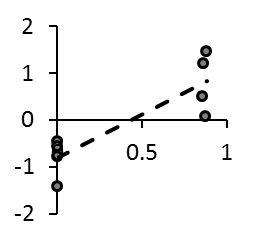 |
|  | **F10:** Waddesdon - 2013* | **F11:** Stanley, et al. ^9^ | **F12:** Morandin and Winston ^10^ |
|  | 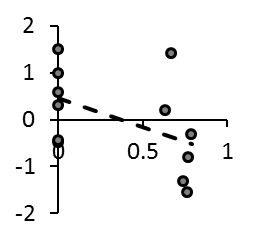 | 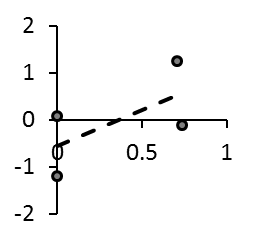 | 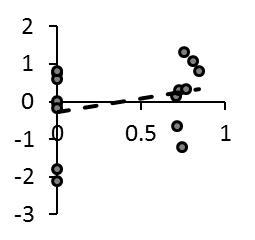 |
|  | **Functional divergence** | | |

| **Yield (Mean=0, SD=1)** | **F13:** Morandin and Winston ^10^ | **F14:** Morandin and Winston ^10^ 2002 expt. | **F15:** Morandin and Winston ^10^ 2003 expt. |
| --- | --- | --- | --- |
|  | 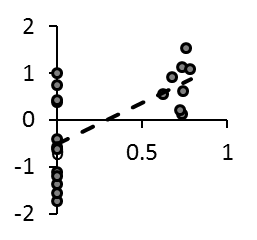 | 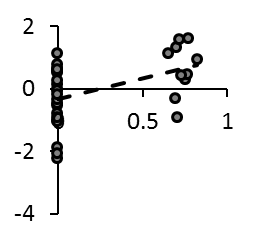 | 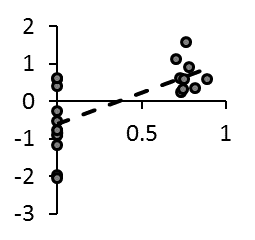 |
|  | **F16:** Zou, et al. ^11^ |  |  |
|  | 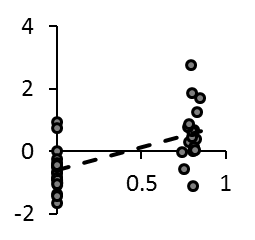 |  |  |
|  | **Functional divergence** | | |

**Supplementary Figure 1d. Individual study raw functional divergence – yield scatter plots.** Scatter plots showing raw data relationship between yield (corrected SD=1) and pollinator functional divergence for individual studies. This represents uncorrected raw measures of functional divergence before the corrections presented in the paper were applied. Note correlations for the scaled functional divergence metric (see methods) and functional divergence derived after excluding control plots (e.g. zero abundance plots with pollinator exclusion cages) are shown in subsequent panels. Sub-panels refer to the individual studies of F1: Lindström, et al. ^6^; F2: Lindström, et al. ^6^; F3: Bommarco, et al. ^7^; F4: Wessex – DK Cabernet; F5: Wessex – PR46W21; F6: Hillesden – 2014 Excalibur; F7: Salisbury – 2012 DK Cabernet; F8: Woodcock, et al. ^8^ NK Molten; F9: Woodcock, et al. ^8^ DK Cabernet; F10: Waddesdon – 2013 Dimension; F11: Stanley, et al. ^9^; F12: Morandin and Winston ^10^ cv45A71; F13: Morandin and Winston ^10^ cvCL289; F14: Morandin and Winston ^10^ 2002 expt. cvDK3235; F15: Morandin and Winston ^10^ 2003 expt. cvDK3235; F16: Zou, et al. ^11^.

| **Yield (Mean=0, SD=1)** | **F1:** Lindström, et al. ^6^ | **F2:** Lindström, et al. ^6^ | **F3:** Bommarco, et al. ^7^ |
| --- | --- | --- | --- |
|  | 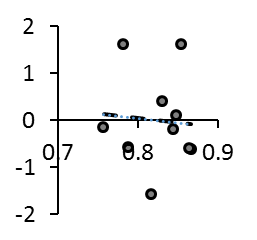 | 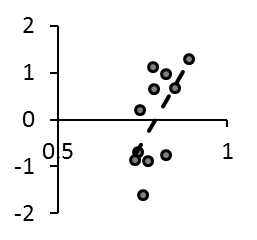 | 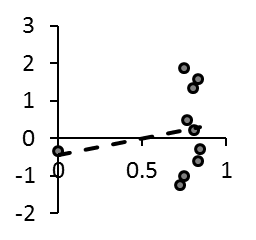 |
|  | **F6:** Hillesden - 2014* | **F7:** Salisbury - 2012* | **F9:** Woodcock, et al. ^8^ |
|  | 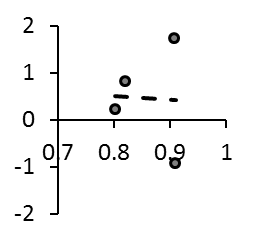 | 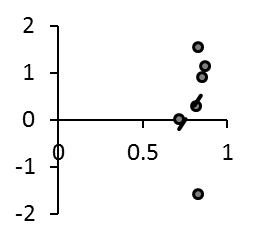 | 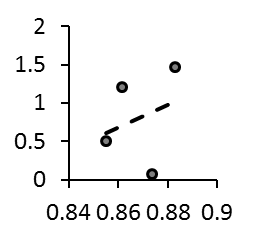 |
|  | **F10:** Waddesdon - 2013* | **F12:** Morandin and Winston ^10^ | **F13:** Morandin and Winston ^10^ |
|  | 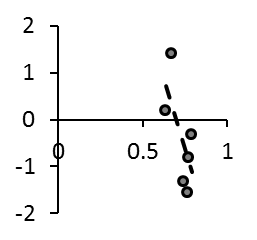 | 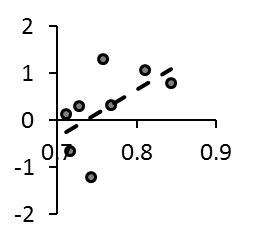 | 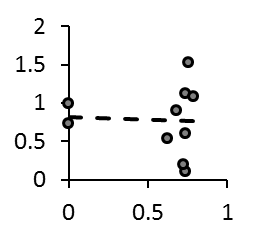 |
|  | **F14:** Morandin and Winston ^10^ 2002 expt. | **F15:** Morandin and Winston ^10^ 2003 expt. | **F16:** Zou, et al. ^11^ |
|  | 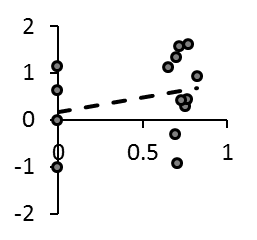 | 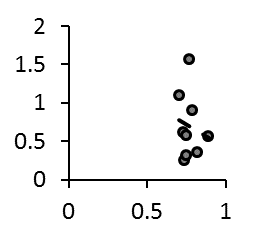 | 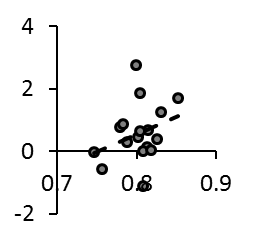 |
|  | **Functional divergence** | | |

**Supplementary Figure 1e. Individual study functional divergence – yield scatter plots.** Scatter plots showing raw data relationship between yield (corrected SD=1) and pollinator functional divergence for individual studies. This represents uncorrected raw measures of functional divergence before the corrections presented in the paper were applied. However, here control plots (i.e. where pollinator exclusion cages were used) have been excluded in the derivation of the correlation. Sub-panels refer to the individual studies of F1: Lindström, et al. ^6^; F2: Lindström, et al. ^6^; F3: Bommarco, et al. ^7^; F6: Hillesden – 2014 Excalibur; F7: Salisbury – 2012 DK Cabernet; F9: Woodcock, et al. ^8^ DK Cabernet; F10: Waddesdon – 2013 Dimension; F12: Morandin and Winston ^10^ cv45A71; F13: Morandin and Winston ^10^ cvCL289; F14: Morandin and Winston ^10^ 2002 expt. cvDK3235; F15: Morandin and Winston ^10^ 2003 expt. cvDK3235; F16: Zou, et al. ^11^. Note the exclusion of controls meant that for some studies there were insufficient replicates for inclusion in the meta-analyses (*N*<4). These are not presented.

| **Yield (Mean=0, SD=1)** | **F1:** Lindström, et al. ^6^ | **F2:** Lindström, et al. ^6^ | **F3:** Bommarco, et al. ^7^ |
| --- | --- | --- | --- |
|  | 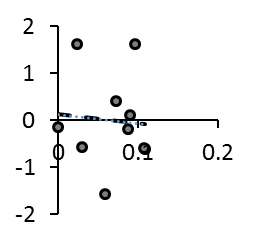 | 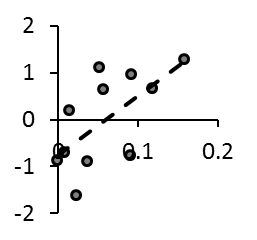 | 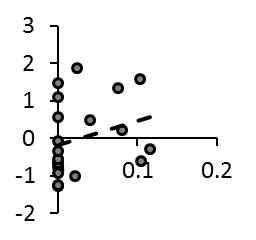 |
|  | **F4:** Wessex - 2013* | **F5:** Wessex - 2013* | **F6:** Hillesden - 2014* |
|  | 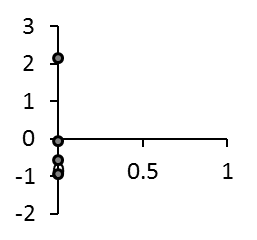 | 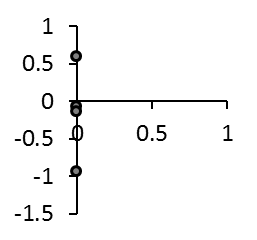 | 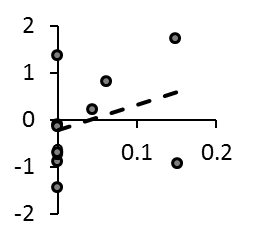 |
|  | **F7:** Salisbury - 2012* | **F8:** Woodcock, et al. ^8^ | **F9:** Woodcock, et al. ^8^ |
|  | 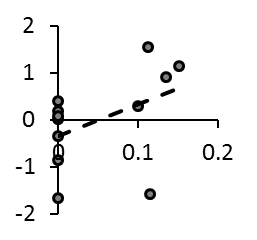 | 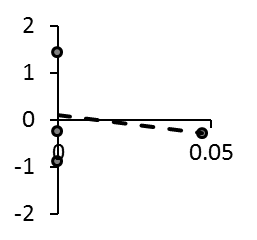 | 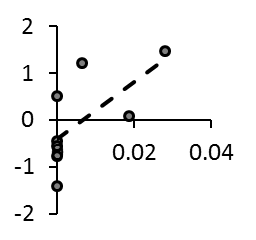 |
|  | **F10:** Waddesdon - 2013* | **F11:** Stanley, et al. ^9^ | **F12:** Morandin and Winston ^10^ |
|  | 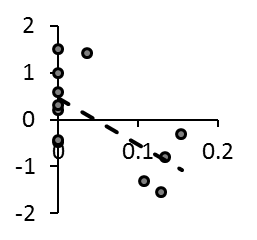 |  |  |
|  | **Functional divergence** | | |

| **Yield (Mean=0, SD=1)** | **F13:** Morandin and Winston ^10^ | **F14:** Morandin and Winston ^10^ 2002 expt. | **F15:** Morandin and Winston ^10^ 2003 expt. |
| --- | --- | --- | --- |
|  |  |  |  |
|  | **F16:** Zou, et al. ^11^ |  |  |
|  |  |  |  |
|  | **Functional divergence** | | |

**Supplementary Figure 1f. Individual study functional divergence – yield scatter plots.** Scatter plots showing raw data relationship between yield (corrected SD=1) and pollinator functional divergence for individual studies. This represents a corrected measure of Functional Divergence where for each study all values of FDiv>0 were rescaled to be equal to the FDiv value - lowest non-zero FDiv value. Sub-panels refer to the individual studies of F1: Lindström, et al. ^6^; F2: Lindström, et al. ^6^; F3: Bommarco, et al. ^7^; F4: Wessex – DK Cabernet; F5: Wessex – PR46W21; F6: Hillesden – 2014 Excalibur; F7: Salisbury – 2012 DK Cabernet; F8: Woodcock, et al. ^8^ NK Molten; F9: Woodcock, et al. ^8^ DK Cabernet; F10: Waddesdon – 2013 Dimension; F11: Stanley, et al. ^9^; F12: Morandin and Winston ^10^ cv45A71; F13: Morandin and Winston ^10^ cvCL289; F14: Morandin and Winston ^10^ 2002 expt. cvDK3235; F15: Morandin and Winston ^10^ 2003 expt. cvDK3235; F16: Zou, et al. ^11^.

| **Yield (Mean=0, SD=1)** | **F1:** Lindström, et al. ^6^ | **F2:** Lindström, et al. ^6^ | **F3:** Bommarco, et al. ^7^ |
| --- | --- | --- | --- |
|  |  |  |  |
|  | **F4:** Wessex - 2013* | **F5:** Wessex - 2013* | **F6:** Hillesden - 2014* |
|  |  |  |  |
|  | **F7:** Salisbury - 2012* | **F8:** Woodcock, et al. ^8^ | **F9:** Woodcock, et al. ^8^ |
|  |  |  |  |
|  | **F10:** Waddesdon - 2013* | **F11:** Stanley, et al. ^9^ | **F12:** Morandin and Winston ^10^ |
|  |  |  |  |
|  | **Body length CWM** | | |

| **Yield (Mean=0, SD=1)** | **F13:** Morandin and Winston ^10^ | **F14:** Morandin and Winston ^10^ 2002 expt. | **F15:** Morandin and Winston ^10^ 2003 expt. |
| --- | --- | --- | --- |
|  |  |  |  |
|  | **F16:** Zou, et al. ^11^ |  |  |
|  |  |  |  |
|  | **Body length CWM** | | |

**Supplementary Figure 1g. Individual study CWM body length – yield scatter plots.** Scatter plots showing raw data relationship between yield (corrected SD=1) and pollinator community weighted mean values (CWM) for body length for individual studies. We only present graphs for CWM values directly tested in the meta-analysis or those present in > 25% of the ΔAIC≤2 sub-set determined from the general linear mixed models assessing additive trait effects on yield. Sub-panels refer to the individual studies of F1: Lindström, et al. ^6^; F2: Lindström, et al. ^6^; F3: Bommarco, et al. ^7^; F4: Wessex – DK Cabernet; F5: Wessex – PR46W21; F6: Hillesden – 2014 Excalibur; F7: Salisbury – 2012 DK Cabernet; F8: Woodcock, et al. ^8^ NK Molten; F9: Woodcock, et al. ^8^ DK Cabernet; F10: Waddesdon – 2013 Dimension; F11: Stanley, et al. ^9^; F12: Morandin and Winston ^10^ cv45A71; F13: Morandin and Winston ^10^ cvCL289; F14: Morandin and Winston ^10^ 2002 expt. cvDK3235; F15: Morandin and Winston ^10^ 2003 expt. cvDK3235; F16: Zou, et al. ^11^.

| **Yield (Mean=0, SD=1)** | **F1:** Lindström, et al. ^6^ | **F2:** Lindström, et al. ^6^ | **F3:** Bommarco, et al. ^7^ |
| --- | --- | --- | --- |
|  |  |  |  |
|  | **F4:** Wessex - 2013* | **F5:** Wessex - 2013* | **F6:** Hillesden - 2014* |
|  |  |  |  |
|  | **F7:** Salisbury - 2012* | **F8:** Woodcock, et al. ^8^ | **F9:** Woodcock, et al. ^8^ |
|  |  |  |  |
|  | **F10:** Waddesdon - 2013* | **F11:** Stanley, et al. ^9^ | **F12:** Morandin and Winston ^10^ |
|  |  |  |  |
|  | **Stigmal contact CWM** | | |

| **Yield (Mean=0, SD=1)** | **F13:** Morandin and Winston ^10^ | **F14:** Morandin and Winston ^10^ 2002 expt. | **F15:** Morandin and Winston ^10^ 2003 expt. |
| --- | --- | --- | --- |
|  |  |  |  |
|  | **F16:** Zou, et al. ^11^ |  |  |
|  |  |  |  |
|  | **Stigmal contact CWM** | | |

**Supplementary Figure 1h. Individual study CWM stigmal contact – yield scatter plots.** Scatter plots showing raw data relationship between yield (corrected SD=1) and pollinator community weighted mean values (CWM) for stigmal contact for individual studies. We only present graphs for CWM values directly tested in the meta-analysis or those present in > 25% of the ΔAIC≤2 sub-set determined from the general linear mixed models assessing additive trait effects on yield. Sub-panels refer to the individual studies of F1: Lindström, et al. ^6^; F2: Lindström, et al. ^6^; F3: Bommarco, et al. ^7^; F4: Wessex – DK Cabernet; F5: Wessex – PR46W21; F6: Hillesden – 2014 Excalibur; F7: Salisbury – 2012 DK Cabernet; F8: Woodcock, et al. ^8^ NK Molten; F9: Woodcock, et al. ^8^ DK Cabernet; F10: Waddesdon – 2013 Dimension; F11: Stanley, et al. ^9^; F12: Morandin and Winston ^10^ cv45A71; F13: Morandin and Winston ^10^ cvCL289; F14: Morandin and Winston ^10^ 2002 expt. cvDK3235; F15: Morandin and Winston ^10^ 2003 expt. cvDK3235; F16: Zou, et al. ^11^.

| **Yield (Mean=0, SD=1)** | **F1:** Lindström, et al. ^6^ | **F2:** Lindström, et al. ^6^ | **F3:** Bommarco, et al. ^7^ |
| --- | --- | --- | --- |
|  |  |  |  |
|  | **F4:** Wessex - 2013* | **F5:** Wessex - 2013* | **F6:** Hillesden - 2014* |
|  |  |  |  |
|  | **F7:** Salisbury - 2012* | **F8:** Woodcock, et al. ^8^ | **F9:** Woodcock, et al. ^8^ |
|  |  |  |  |
|  | **F10:** Waddesdon - 2013* | **F11:** Stanley, et al. ^9^ | **F12:** Morandin and Winston ^10^ |
|  |  |  |  |
|  | **Hairiness index CWM** | | |

| **Yield (Mean=0, SD=1)** | **F13:** Morandin and Winston ^10^ | **F14:** Morandin and Winston ^10^ 2002 expt. | **F15:** Morandin and Winston ^10^ 2003 expt. |
| --- | --- | --- | --- |
|  |  |  |  |
|  | **F16:** Zou, et al. ^11^ |  |  |
|  |  |  |  |
|  | **Hairiness index CWM** | | |

**Supplementary Figure 1i. Individual study CEM hairiness index – yield scatter plots.** Scatter plots showing raw data relationship between yield (corrected SD=1) and pollinator community weighted mean values (CWM) for the hairiness index for individual studies. We only present graphs for CWM values directly tested in the meta-analysis or those present in > 25% of the ΔAIC≤2 sub-set determined from the general linear mixed models assessing additive trait effects on yield. Sub-panels refer to the individual studies of F1: Lindström, et al. ^6^; F2: Lindström, et al. ^6^; F3: Bommarco, et al. ^7^; F4: Wessex – DK Cabernet; F5: Wessex – PR46W21; F6: Hillesden – 2014 Excalibur; F7: Salisbury – 2012 DK Cabernet; F8: Woodcock, et al. ^8^ NK Molten; F9: Woodcock, et al. ^8^ DK Cabernet; F10: Waddesdon – 2013 Dimension; F11: Stanley, et al. ^9^; F12: Morandin and Winston ^10^ cv45A71; F13: Morandin and Winston ^10^ cvCL289; F14: Morandin and Winston ^10^ 2002 expt. cvDK3235; F15: Morandin and Winston ^10^ 2003 expt. cvDK3235; F16: Zou, et al. ^11^.

| **Yield (Mean=0, SD=1)** | **F1:** Lindström, et al. ^6^ | **F2:** Lindström, et al. ^6^ | **F3:** Bommarco, et al. ^7^ |
| --- | --- | --- | --- |
|  |  |  |  |
|  | **F4:** Wessex - 2013* | **F5:** Wessex - 2013* | **F6:** Hillesden - 2014* |
|  |  |  |  |
|  | **F7:** Salisbury - 2012* | **F8:** Woodcock, et al. ^8^ | **F9:** Woodcock, et al. ^8^ |
|  |  |  |  |
|  | **F10:** Waddesdon - 2013* | **F11:** Stanley, et al. ^9^ | **F12:** Morandin and Winston ^10^ |
|  |  |  |  |
|  | **CWM propodeal corbicula** | | |

| **Yield (Mean=0, SD=1)** | **F13:** Morandin and Winston ^10^ | **F14:** Morandin and Winston ^10^ 2002 expt. | **F15:** Morandin and Winston ^10^ 2003 expt. |
| --- | --- | --- | --- |
|  |  |  |  |
|  | **F16:** Zou, et al. ^11^ |  |  |
|  |  |  |  |
|  | **CWM propodeal corbicula** | | |

**Supplementary Figure 1j. Individual study CWM propodeal corbicula – yield scatter plots.** Scatter plots showing raw data relationship between yield (corrected SD=1) and pollinator community weighted mean values (CWM) for the presence of a propodeal corbicula for individual studies. We only present graphs for CWM values directly tested in the meta-analysis or those present in > 25% of the ΔAIC≤2 sub-set determined from the general linear mixed models assessing additive trait effects on yield. Sub-panels refer to the individual studies of F1: Lindström, et al. ^6^; F2: Lindström, et al. ^6^; F3: Bommarco, et al. ^7^; F4: Wessex – DK Cabernet; F5: Wessex – PR46W21; F6: Hillesden – 2014 Excalibur; F7: Salisbury – 2012 DK Cabernet; F8: Woodcock, et al. ^8^ NK Molten; F9: Woodcock, et al. ^8^ DK Cabernet; F10: Waddesdon – 2013 Dimension; F11: Stanley, et al. ^9^; F12: Morandin and Winston ^10^ cv45A71; F13: Morandin and Winston ^10^ cvCL289; F14: Morandin and Winston ^10^ 2002 expt. cvDK3235; F15: Morandin and Winston ^10^ 2003 expt. cvDK3235; F16: Zou, et al. ^11^.

| **Yield (Mean=0, SD=1)** | **F1:** Lindström, et al. ^6^ | **F2:** Lindström, et al. ^6^ | **F3:** Bommarco, et al. ^7^ |
| --- | --- | --- | --- |
|  |  |  |  |
|  | **F4:** Wessex - 2013* | **F5:** Wessex - 2013* | **F6:** Hillesden - 2014* |
|  |  |  |  |
|  | **F7:** Salisbury - 2012* | **F8:** Woodcock, et al. ^8^ | **F9:** Woodcock, et al. ^8^ |
|  |  |  |  |
|  | **F10:** Waddesdon - 2013* | **F11:** Stanley, et al. ^9^ | **F12:** Morandin and Winston ^10^ |
|  |  |  |  |
|  | **CWM long tongue** | | |

| **Yield (Mean=0, SD=1)** | **F13:** Morandin and Winston ^10^ | **F14:** Morandin and Winston ^10^ 2002 expt. | **F15:** Morandin and Winston ^10^ 2003 expt. |
| --- | --- | --- | --- |
|  |  |  |  |
|  | **F16:** Zou, et al. ^11^ |  |  |
|  |  |  |  |
|  | **CWM long tongue** | | |

**Supplementary Figure 1k. Individual study CEM tongue length – yield scatter plots.** Scatter plots showing raw data relationship between yield (corrected SD=1) and pollinator community weighted mean values (CWM) for tongue length for individual studies. We only present graphs for CWM values directly tested in the meta-analysis or those present in > 25% of the ΔAIC≤2 sub-set determined from the general linear mixed models assessing additive trait effects on yield. Note these relationships are only included for the interpretation of the GLMM analysis of additive effects of multiple traits on yield and are note relevant for the main meta-analysis). Tongue length is binary (long =1, other=0). Sub-panels refer to the individual studies of F1: Lindström, et al. ^6^; F2: Lindström, et al. ^6^; F3: Bommarco, et al. ^7^; F4: Wessex – DK Cabernet; F5: Wessex – PR46W21; F6: Hillesden – 2014 Excalibur; F7: Salisbury – 2012 DK Cabernet; F8: Woodcock, et al. ^8^ NK Molten; F9: Woodcock, et al. ^8^ DK Cabernet; F10: Waddesdon – 2013 Dimension; F11: Stanley, et al. ^9^; F12: Morandin and Winston ^10^ cv45A71; F13: Morandin and Winston ^10^ cvCL289; F14: Morandin and Winston ^10^ 2002 expt. cvDK3235; F15: Morandin and Winston ^10^ 2003 expt. cvDK3235; F16: Zou, et al. ^11^.

| **Yield (Mean=0, SD=1)** | **F1:** Lindström, et al. ^6^ | **F2:** Lindström, et al. ^6^ | **F3:** Bommarco, et al. ^7^ |
| --- | --- | --- | --- |
|  |  |  |  |
|  | **F4:** Wessex - 2013* | **F5:** Wessex - 2013* | **F6:** Hillesden - 2014* |
|  |  |  |  |
|  | **F7:** Salisbury - 2012* | **F8:** Woodcock, et al. ^8^ | **F9:** Woodcock, et al. ^8^ |
|  |  |  |  |
|  | **F10:** Waddesdon - 2013* | **F11:** Stanley, et al. ^9^ | **F12:** Morandin and Winston ^10^ |
|  |  |  |  |
|  | **Phylogenetic MPD** | | |

| **Yield (Mean=0, SD=1)** | **F13:** Morandin and Winston ^10^ | **F14:** Morandin and Winston ^10^ 2002 expt. | **F15:** Morandin and Winston ^10^ 2003 expt. |
| --- | --- | --- | --- |
|  |  |  |  |
|  | **F16:** Zou, et al. ^11^ |  |  |
|  |  |  |  |
|  | **Phylogenetic MPD** | | |

**Supplementary Figure 1l. Individual study phylogenetic MPD – yield scatter plots.** Scatter plots showing raw data relationship between yield (corrected SD=1) and pollinator community weighted mean values (CWM) for phylogenetic mean pairwise distance (MPD) for individual studies. Sub-panels refer to the individual studies of F1: Lindström, et al. ^6^; F2: Lindström, et al. ^6^; F3: Bommarco, et al. ^7^; F4: Wessex – DK Cabernet; F5: Wessex – PR46W21; F6: Hillesden – 2014 Excalibur; F7: Salisbury – 2012 DK Cabernet; F8: Woodcock, et al. ^8^ NK Molten; F9: Woodcock, et al. ^8^ DK Cabernet; F10: Waddesdon – 2013 Dimension; F11: Stanley, et al. ^9^; F12: Morandin and Winston ^10^ cv45A71; F13: Morandin and Winston ^10^ cvCL289; F14: Morandin and Winston ^10^ 2002 expt. cvDK3235; F15: Morandin and Winston ^10^ 2003 expt. cvDK3235; F16: Zou, et al. ^11^.

**Supplementary Methods. Meta-analysis diagnostics**.

Funnel plots and Cook’s distance diagnostic plots for each of the considered measures of invertebrate community structure used to predict oilseed rape yields. These were determined in the metaphor package implemented in R 3.5.0. In cases where studies were identified as having an undue influence on parameter estimates the analyses were repeated after the exclusion of this data point.

| **Study** | **Cook’s distance** |  |
| --- | --- | --- |
|  | **N (meso)** | **SR (Field)** |
| **Garratt, et al. ^4^** | 0.015 | 0.164 |
| **Jauker and Wolters ^1^** | 0.001 | 0.203 |
| **Jauker, et al. ^2^** | **2.435** | **10.991** |
| **Soroka, et al. ^5^-**Yr’94 | 0.059 | 0.374 |
| **Soroka, et al. ^5^-**Yr’95 | 0.097 | 0.005 |
| **Steffan-Dewenter ^3^ –** Express MSL | **1.313** | **1.454** |
| **Steffan-Dewenter ^3^ –** Express | 0.003 | 0.378 |

The above table gives summary Cook’s distance measurements for meta-analyses relating to data sets originating from mesocosm studies. We used Cook’s distance to identify individual studies that had a large influence on the estimates derived from the meta-analysis, where a threshold for high influence was set at 1 ^12^. In these cases the high influence studies were removed and from analysis data set. N=Abundance, SR=species richness

| **Study** |  | | **Cook’s distance** | | | | | | |  |
| --- | --- | --- | --- | --- | --- | --- | --- | --- | --- | --- |
|  | **N** | **SR** | | **EGR** | **FDiv-no controls** | **FDiv-scaled** | **BL-cwm** | **SC -cwm** | **HI -cwm** | **MPD** |
| **Hillesden (2014)*** | 0.002 | **1.119** | | 0.828 | 0.267 | 2.025 | 0.803 | 1.508 | 0.763 | 0.856 |
| **Lindström, et al. ^6^ - Excalibur** | 0.110 | 0.449 | | NA | 0.035 | 0.075 | 1.143 | 1.257 | 1.321 | 1.843 |
| **Lindström, et al. ^6^ - Galileo** | 0.023 | 0.009 | | 0.296 | 0.36 | 0.266 | 0.001 | 0.042 | 0.002 | 0.034 |
| **Wessex (2013) -** DKCabernet | 0.002 | 0.007 | | 0.001 | NA | 0.029 | 0.02 | 0.025 | 0.014 | 0.008 |
| **Wessex (2013) -** PR46W21 | 0.049 | 0.08 | | 0.111 | NA | 0.001 | 0.024 | 0.01 | 0.034 | 0.072 |
| **Stanley, et al. ^9^** | 0.002 | 0.001 | | 0.001 | NA | 0.179 | 0.002 | 0.001 | 0.002 | 0.001 |
| **Salisbury (2012)** – DKCabernet | 0.008 | 0.003 | | 0.009 | 0.001 | 0.014 | 0.001 | 0.001 | 0.001 | 0.003 |
| **Woodcock, et al. ^8^** – DKCabernet | 0.157 | 0.005 | | 0.427 | 0.003 | 4.418 | 0.156 | 0.177 | 0.591 | 0.319 |
| **Woodcock, et al. ^8^ –** NKMolten | 0.001 | 0.001 | | 0.001 | NA | 0.208 | 0.001 | 0.001 | 0.001 | 0.001 |
| **Waddesdon (2013)*** | 0.252 | 0.766 | | 1.179 | 0.630 | 0.315 | 0.002 | 0.143 | 0.004 | 0.028 |
| **Zou, et al. ^11^** | 0.006 | 0.001 | | 0.103 | 0.021 | 0.02 | 0.068 | 0.014 | 0.006 | 0.085 |
| **Bommarco, et al. ^7^** | 0.033 | 0.163 | | 0.08 | 0.162 | 0.069 | 0.732 | 0.854 | 0.649 | 0.644 |
| **Morandin and Winston ^10^-** Advanta cv45A71 -2002 | 0.001 | 0.015 | | 0.008 | 0.061 | 0.146 | 0.180 | 0.145 | 0.169 | 0.027 |
| **Morandin and Winston ^10^-** cvDK3235 - 2002 | 0.001 | 0.213 | | 0.045 | 4.765 | 0.022 | 0.320 | 0.607 | 0.266 | 0.375 |
| **Morandin and Winston ^10^-** Advanta cvCL289 -2003 | 0.151 | 0.24 | | 0.425 | 1.169 | 2.025 | 1.288 | 1.735 | 1.439 | 0.581 |
| **Morandin and Winston ^10^-** cvDK3235 - 2003 | 0.003 | 0.067 | | 0.014 | 1.407 | 0.075 | 0.100 | 0.189 | 0.083 | 0.117 |

The above table gives summary Cook’s distance measurements for meta-analyses relating to Fig. 2 in the main paper. We used Cook’s distance to identify individual studies that had a large influence on the estimates derived from the meta-analysis, where a threshold for high influence was set at 1 ^12^ – these values are underlined. Analyses were then repeated after these data points were removed. Where N=Abundance, SR=species richness, EGR=effect group richness, FDiv-no control=functional divergence excluding plots where controls were present, FDIV-scaled=scaled functional divergence, BL-cwm=body length CWM, SC-cwm=Stigmal contact behaviour CWM, and HI-cwm=Hairiness index CWM, MPD=Mean phylogenetic pairwise distance. Note that NA values indicate there was insufficient variation within a data set to derive a correlation coefficient between the yield metric and the measure of pollinator community structure for the original meta-analysis.

**Funnel plots for overall relationship between metrics of pollinator community structure and yield. All funnel plots are for final analyses following the removal of outliers with high influence (Cook’s distance>1).**

**Supplementary Methods. Pearson’s correlations between effect traits and the mean pollen stigmal deposition**.

Pearson’s correlations between proposed morphological or behavioural effect traits (Supplementary Table 2) and the mean pollen stigmal deposition recorded for insects foraging on *Brassica rapa*, a close relative of oilseed rape (*Bassica napa*). This data was derived from published values given by Howlett, et al. ^13^ and Rader, et al. ^14^. Pollen stigmal deposition rates represent a directly measured effect trait, and while they are likely to vary in both space and time they provide a valuable base line approach for identifying key effects traits that support pollination services. However, due to the time consuming nature of their collection are typically collected for only a small number of species of pollinators (normally the most abundant ones). Only nine species in our data set had any viable published data on pollen deposition rates. In several cases it was necessary to use data on closely related species (as indicated in the table below). Although these issues introduce clear caveats, this approach provides a base line validation for the importance of a specific trait in promoting increased seed set in oilseed rape. As such they allow the derivation of trait community weighted means for the main meta-analysis to be restricted to those traits with the greatest evidence for being true effect traits affecting pollination success in oilseed rape. It was only possible to test for correlations between a limited number of our derived effects traits and the pollen stigmal depositions rates, as for the 9 species considered many traits either no or very low variation. In these cases no assessment of the traits importance could be made, and as such they were ignored in subsequent assessments of the mass ratio hypothesis using community weighted trait means.

| **Species** | **Stigmal deposition rates (SDR)** |  | **Body length mm** | **Time foraging on flower (seconds)** | **Nectar foraging** | **Pollen foraging** | **Stigmal contact when foraging** | **Dry pollen on body** | **Body Hairiness index** |
| --- | --- | --- | --- | --- | --- | --- | --- | --- | --- |
| *Apis mellifera (Apidae)* | 5768 |  | 16.5 | 3.66 | 0.98 | 0.06 | 0.4 | 0.78 | 0.92 |
| *Bombus terrestris (Apidae)* | 4114 |  | 20 | 2.9 | 0.97 | 0.08 | 0.85 | 0.42 | 0.88 |
| *Lassiglossum spp (*Halictidae) | 1108 |  | 6.6 | 12.9 | 0.97 | 0.75 | 0.69 | 0.79 | 0.67 |
| *Bibio marci* ^†^ (Bibionidae) | 4872 |  | 12 | 14.7 | 0.37 | 0.19 | 0.37 | 0.19 | 0.59 |
| *Eristalis* sp (Syrphidae) | 4357 |  | 15.3 | 5.0 | 0.31 | 0.77 | 0.62 | 0.47 | 0.62 |
| *Melanostoma* sp (Syrphidae) | 1525 |  | 8.3 | 5.0 | 0.46 | 0.64 | 0.19 | 0.1 | 0.25 |
| *Syrphus ribesii* ^††††^ (Syrphidae) | 1212 |  | 11.1 | 7.7 | 0.89 | 0.12 | 0.12 | 0.34 | 0.17 |
| Calypterate fly ^†††^ (Diptera) | 1813 |  | 7.1 | 20.4 | 0.14 | 0.67 | 0.27 | 0.07 | 0.17 |
| *Chloromyia* sp ^††††^ (Stratomyidae) | 2076 |  | 9.2 | 6.0 | 0.75 | 0.5 | 0.5 | 0.5 | 0.17 |
| Pearsons corr. coef. |  |  | 0.87 | -0.39 | 0.25 | -0.44 | 0.70 | 0.23 | 0.71 |
| Significance |  |  | **T_7_=4.78, p=0.002** | T_7_=-1.13, p>0.05 | T_7_=0.71, p>0.05 | T_7_=-1.31, p>0.05 | **T_7_=2.61, p=0.03** | T_7_=063, p>0.05 | **T_7_=2. 66, p=0.03** |

^†^ using data on SDR from closely related *Dilophus* sp (Bibionidae)

^††^ using data on SDR from closely related *Melangyna* sp, also in Syrphini (Syrphidae)

^†††^ using data for *Calliphora* sp (Calliphodidae)

^††††^ using data on *Odontomyia* sp, a con-familial member of the Stratomyiidae

**Supplementary Methods. PRISMA flow diagram showing the process of study selection for the meta-analysis.**

**Records identified through database searching**

[Web of Science Search – TS=(pollination OR Pollinators OR Pollinator) AND TS=(oilseed rape OR brassica napus OR rapeseed OR canola) AND TS=(yield)]. WOS search from 1980 – March 2018

(n = 138)

**Additional records identified through other sources**
(n = 7)

## Identification

**Records after duplicates removed**
(n = 145)

## Included

**Studies included in quantitative synthesis (meta-analysis)**(n = 18 , where Mesocosm n=5; Field based n=10).

These were separated by year and variety of oilseed rape into 23 studies (Mesocosm n=7; Field based n=16)

**Studies included in qualitative synthesis**
(n = 18, where Mesocosm n=5; Field based n=10). (Mesocosm n=7; Field based n=16)

**Full-text articles excluded, with reasons**
(n = 127)

**Records excluded**
(n = 0)

## Screening

## Eligibility

**Records screened**
(n = 145)

**Full-text articles assessed for eligibility.**

Eligibility requirements: a) community level data on pollinator species; b) measure of yield of oilseed rape; c) Samples from ≥ 4 experimental units (field or mesocosm);
(n = )

**Supplementary Methods. Summary methodologies of unpublished studies describing the sampling of pollinator communities and the assessment of oilseed rape yield in response to pollination.**

**Study name ‘Wessex (2013)’**

**Pollinator communities:** Pollinator visit data were collected from winter sown oilseed rape fields in southern England (NW corner 51.415482^o^N, -2.2892761^o^W; SE corner 51.087135^o^N, -1.5037537^o^W) for eight fields winter sown with the oilseed rape varieties DK Cabernet (2 fields), PR46W21 (2 fields), Fashion (1 Field), Pioneer44 (1 Field) and Excellium (1 Field). This was undertaken in May-June 2013. Each field contained three 58m transects perpendicular to the centre of the field edge. Survey points were set up at 8m, 33m and 58m distance from the crop edge. At each point a 1m^2^ quadrat was observed for 5 mins and all flower visits recorded (i.e. one individual pollinator could make multiple visits). Surveys were carried out between 10:00 am and 18:00pm; wind speed of Beaufort scale 3 or less; and temperature between 12 and 22^o^C. If weather allowed, each quadrat was surveyed twice on the same date. Flower visitors were identified to species and individuals caught for post survey identification if required. Individual on the wing were identified to Hymenoptera: *Apis mellifera*, *Bombus* spp. or morphotype for other groups. **Oilseed rape yield:** Yield data were assessed concurrent with the pollinator surveys. To assess yield in relation to insect pollination two plants at each of the 5 sampling points were marked before flowering with plastic plant labels attached with plant tie. Plants were selected to be of approximately the same size and phenological stage. Plants were allocated at random to be either left as open pollinated or covered with a micro perforated pollination bag (Focus Packaging& Design Ltd, Lincolnshire, UK) to prevent insect pollination. Plants were checked during the flowering season (approximately fortnightly) and bags moved up with growing plant parts. Once the plants had ceased flowering, the bags were removed and the plants were left to ripen in situ before harvesting. The seeds from the entire plant were then extracted by hand and put through a seed cleaner, then counted with an automated seed counter (Elmor AG, Elmor Ltd, Switzerland). Full commercial agronomic inputs were applied to the oilseed rape to maximise yield.

**Study name ‘Salisbury Plane (2012)’**

**Pollinator communities:** Pollinator communities were assessed in three fields of oilseed rape (DK Cabernet variety) from each of two farms in Wiltshire, UK. These were Windwhistle Farm (N 51.0533333^o^, W -1.8916667^o^) and Burcombe Manor (N 51.078333 ^o^; W -1.901666 ^o^). For each of the six fields, two separate 50 m × 2 m fixed transects were established from the edge along tram lines. Each transect was started at a distance of 25 m from the crop margin, with paired transects within individual fields separated by 22 m (the width of the tram lines). For two month (29/4/2012 to 31/5/2012) over the flowering period of oilseed rape, individual transect were surveyed for bees on eight separate occasions following standard limits for weather conditions for butterfly surveys given by Pollard and Yates ^15^. As the sampling season was relatively early transects were walked between 10.30 - 16.00 hours to ensure high levels of bee activity. Each transect was walked for a period of 30 minutes, so that a single field (the experimental unit) received 8 hours of observations on a 100 × 2 m area (equivalent to 2.4 minutes m^-2^). All bees, hoverflies and butterflies were identified to species, although in some occasions either generic level identifications (*Lasiglossum* spp.) were used. All other flies were either identified to species (*Bibio marci*) or functional type (Delia sp.). Note the approaches for assessing the pollinator communists are described in Woodcock, et al. ^16^ although assessments of yield were not presented in that paper. **Oilseed rape yield:** 2.5 × 13 m fine net pollinator exclusion cages constructed from 0.6 mm mesh were extended over aluminium frames. These were established on each of the six fields described above. All cages were 1.8 m in height to allow sufficient height for the full phenological development of the oilseed rape, while being low enough so that the boom height of the farm pesticide sprayer can pass over it. In all cases pollinator exclusion cages will erected just prior to crop flowering (March 26-30^th^ 2012). Trials demonstrated that the key fungicide spraying operations (Pernezyn for the control of Sclerotina) as undertaken in the early-mid flowering period can pass through this netting when applied at c. 2.5 bar pressure. Solid pellet nitrogen was applied by hand within exclusion cages at the same rate as the rest of the field. Following the end of flowering the cages were removed and the crop was left to develop as normal until the point of normal agronomic harvest. A trials combine harvester was then used to collect a central 2 × 10 m strip of crop in the exclusion cages and a comparable areas outside the cage (this non caged area would have been exposed to pollinators). Seed was cleaned and weighed to produce a yield in tonnes ha^-1^.

**Study name ‘Waddesdon (2013)’**

**Pollinator communities:** This study was undertaken in 2013 on winter sown oilseed rape fields on six oilseed rape fields at the Waddesdon Estate in Oxfordshire, UK (N 51.842705^o^, W -0.93724447 ^o^). All fields were sown with the restored hybrid variety Dimension. To assess the population densities of foraging pollinators timed observations (5 minutes) were undertaken on four occasions within open 2 × 2 m areas located at 10, 20 and 50 m along transects running into oilseed rape fields. This occurred during the period of peak oilseed rape flowering in May-June 2013. Observations were undertaken between 10.00 - 16.00 hours following the weather limitations defined by Pollard and Yates ^15^. All bees, hoverflies and butterflies were identified to species, although in some occasions either generic level identifications (*Lasiglossum* spp.) were used. All other flies were either identified to species (*Bibio marci*) or functional type (Delia sp.). **Oilseed rape yield:** Along each transect we assessed the provision of pollination services by quantifying seed set of the oilseed rape crop at of 10, 20 and 50 m. To assess the contribution made by insect pollinators to seed set exclusion cages were used. Each exclusion cage was 1.8 m high and was made from 0.6 mm agricultural netting that prevented access by insect pollinators while allowing inputs of liquid pesticide and fungicide. Solid pellet nitrogen was applied by hand within exclusion cages at the same rate as the rest of the field. Pollinator exclusion cages were erected in early March 2013 when the seedlings were c. 15-20 cm in height. When flowering was complete exclusion cages were removed. Following maturation of the crop in July 2013 the crop was harvested by hand. These samples were oven dried to constant weight at 80°C and then threshed using a Minibatt thresher (GODE, France) to determine a yield in tonnes ha^-1^.

**Study name ‘Hillesden (2014)’**

**Pollinator communities:** This study was undertaken in 2013 on winter sown oilseed rape fields on six oilseed rape fields at the Hillesden Estate in Oxfordshire, UK (N 51.954444^o^, W -1.000277^o^). All oilseed rape fields were winter sown with the restored hybrid variety Excalibur. To assess the population densities of foraging pollinators timed observations (5 minutes) were undertaken on four occasions within open 2 × 10 m areas located at 0-10 m and 45-55 m along transects running into oilseed rape fields. This occurred during the period of peak oilseed rape flowering in May 2014. Observations were undertaken between 10.00 - 16.00 hours following the weather limitations defined by Pollard and Yates (1993). All bees, hoverflies and butterflies were identified to species, although in some occasions either generic level identifications (*Lasiglossum* spp.) were used. All other flies were either identified to species (*Bibio marci*) or functional type (Delia sp.). **Oilseed rape yield:** To assess the contribution made by insect pollinators to seed set exclusion cages were used. A control 2 × 2 m areas of crop (where insect pollinators had full access to the crop) was compared to an adjoining 2 × 2 m areas covered in pollinator exclusion cages. Each exclusion cage was 1.8 m high and was made from 0.6 mm agricultural netting that prevented access by insect pollinators while allowing inputs of liquid pesticide and fungicide. Solid pellet nitrogen was applied by hand within exclusion cages at the same rate as the rest of the field. Pollinator exclusion cages were erected in early March 2013 when the seedlings were c. 15-20 cm in height. We assessed the provision of pollination services within open and caged 2 × 2 m areas at two distances (5 and 50 m) into oilseed rape fields. Following maturation of the crop in July 2013 the crop was harvested by hand. Five plants from each 2 × 2 m area were randomly selected and were oven dried. Seeds were removed by hand and counted to provide a total for each plant using an automated seed counter (Elmor C1 seed counter, Elmor Ltd, Switzerland).

**Supplementary Methods. Definition of effect groups**.

Using the R package ‘pvclust’, Wards algorithm was used to hierarchically cluster species based on a matrix of the 15 defined effect traits. Multiscale bootstrap resampling (1000 iterations) was then used to calculate approximate unbiased (au) p values for each edge (or split) of the cluster. These au p values where then used to cluster species into functional groups using α=0.95 as a threshold for each cluster with the function pv.pick. This produced a total of 5 effect group clusters with three species not allocated to any cluster. For practical reasons these remaining species were arbitrarily aggregated to form a sixth effect group cluster. The following tables give information on both the information on average effect trait values of the 6 effect group clusters as well as the taxonomic composition of each cluster.

| **Trait** | **EG1** | **EG2** | **EG3** | **EG4** | **EG5** | **EG6** |
| --- | --- | --- | --- | --- | --- | --- |
| Length (mm) | 7.10 (SE 0.27) | 9.8 (SE 0.75) | 10.2 (SE 1.25) | 11.0 (SE 0.67) | 17.0 (SE 0.97) | 7.6 (SE 1.34) |
| Time foraging | 17.9 (SE 2.46) | 13.0 (SE 1.15) | 9.90 (SE 0.98) | 6.8 (SE 0.35) | 3.8 (SE 0.27) | 9.2 (SE 1.29) |
| Nectar foraging ^†^ | 0.60 (SE 0.25) | 0.90 (SE 0.09) | 1.00 (SE 0.02) | 0.90 (SE 0.08) | 0.8 (SE 0.05) | 0.90 (SE 0.12) |
| Pollen foraging | 0.80 (SE 0.03) | 0.50 (SE 0.13) | 0.70 (SE 0.08) | 0.40 (SE 0.11) | 0.30 (SE 0.06) | 0.70 (SE 0.33) |
| Stigmal contact | 0.70 (SE 0.21) | 0.80 (SE 0.09) | 0.90 (SE 0.01) | 0.60 (SE 0.14) | 0.90 (SE 0.05) | 1.00 (SE 0.00) |
| Dry pollen on body | 0.50 (SE 0.22) | 0.50 (SE 0.14) | 1.00 (SE 0.06) | 0.50 (SE 0.14) | 0.40 (SE 0.05) | 0.70 (SE 0.33) |
| Hairiness index | 0.60 (SE 0.2) | 0.60 (SE 0.13) | 0.70 (SE 0.11) | 0.50 (SE 0.12) | 0.80 (SE 0.06) | 0.40 (SE 0.18) |
| Corbicula tibiae strict ^†^ | 0.00 (SE 0.0) | 0.20 (SE 0.15) | 0.00 (SE 0.0) | 0.00 (SE 0.0) | 0.80 (SE 0.09) | 0.00 (SE 0.0) |
| Corbicula propodeal ^†^ | 0.00 (SE 0.0) | 0.50 (SE 0.21) | 1.00 (SE 0.00) | 0.30 (SE 0.17) | 0.00 (SE 0.0) | 0.40 (SE 0.34) |
| Corbicula abdomen ^†^ | 0.00 (SE 0.0) | 0.00 (SE 0.0) | 0.00 (SE 0.00) | 0.20 (SE 0.13) | 0.00 (SE 0.0) | 0.40 (SE 0.34) |
| Femoral corbiculae ^†^ | 0.70 (SE 0.34) | 0.50 (SE 0.21) | 0.8 (SE 0.2) | 0.40 (SE 0.19) | 0.10 (SE 0.04) | 0.70 (SE 0.34) |
| Basitarsal scopa | 0.00 (SE 0.00) | 0.00 (SE 0.0) | 0.00 (SE 0.0) | 0.00 (SE 0.0) | 0.10 (SE 0.04) | 0.00 (SE 0.0) |
| Corbicula pollen moist ^†^ | 0.00 (SE 0.00) | 0.00 (SE 0.0) | 0.00 (SE 0.0) | 0.00 (SE 0.0) | 0.80 (SE 0.09) | 0.00 (SE 0.0) |
| Pollen carried in crop ^†^ | 0.00 (SE 0.00) | 0.00 (SE 0.0) | 0.00 (SE 0.0) | 0.00 (SE 0.0) | 0.00 (SE 0) | 0.40 (SE 0.34) |
| Tongue long ^†^ | 0.00 (SE 0.00) | 0.2 (SE 0.15) | 0.00 (SE 0.0) | 0.2 (SE 0.13) | 0.40 (SE 0.09) | 0.40 (SE 0.34) |
| Tongue medium ^†^ | 0.00 (SE 0.00) | 0.00 (SE 0.00) | 0.00 (SE 0.0) | 0.00 (SE 0.0) | 0.50 (SE 0.1) | 0.40 (SE 0.34) |
| Tongue short ^†^ | 1.00 (SE 0.00) | 0.90 (SE 0.15) | 1 (SE 0) | 0.9 (SE 0.13) | 0.20 (SE 0.07) | 0.40 (SE 0.34) |
| Chewing mouthparts ^†^ | 0.00 (SE 0.00) | 0.00 (SE 0.0) | 0.00 (SE 0.0) | 0.00 (SE 0.0) | 0.10 (SE 0.05) | 0.00 (SE 0.0) |

^†^ For these traits the average value represents the probability of a given morphological characteristic being found within species of that effect group.

The above table gives the mean and standard error (in parenthesis) effect trait value for each of the six effect groups (species compositing given in tables 2-7 below). See table 3 main paper for definition of each trait. Note that for ordinal effect traits (e.g. mouthpart structure, which is either long, medium, short tongue as well as chewing mouthparts) dummy variable shave been used (defining a trait a 1 = present, 0=absent) so that a percentage representation of that trait in the effect group cluster can be provided.

Species composition of effect group cluster 1.

| **Taxonomic group** | **Order** | **Family** | **Abbreviation** |
| --- | --- | --- | --- |
| Calypterate fly | Diptera | Anthomyiidae, Muscidae and Caliphodidae | Calypt.fly |
| *Halictus sp* | Hymenoptera | Halticidae | Hal.sp. |
| *Lassiglossum spp* | Hymenoptera | Halticidae | Las.sp. |

Species composition of effect group cluster 2.

| **Taxonomic group** | **Order** | **Family** | **Abbreviation** |
| --- | --- | --- | --- |
| *Andrena dorsata* | Hymenoptera | Andrenidae | And.dor. |
| *Andrena fulva* | Hymenoptera | Andrenidae | And.ful. |
| *Andrena thaspii* | Hymenoptera | Andrenidae | And tha |
| *Bibio marci* | Diptera | Bibionidae | Bib.mar. |
| *Eucera chinensis* | Hymenoptera | Apidae | Euc.chi. |
| *Sphaerophoria spp* | Diptera | Syrphidae | Sphaer.sp. |
| *Syritta pipiens* | Diptera | Syrphidae | Syri.pi. |

Species composition of effect group cluster 3.

| **Taxonomic group** | **Order** | **Family** | **Abbreviation** |
| --- | --- | --- | --- |
| *Andrena haemorhoa* | Hymenoptera | Andrenidae | And.hae. |
| *Andrena nigroaenea* | Hymenoptera | Andrenidae | And.nig. |
| *Andrena sp.* | Hymenoptera | Andrenidae | And.sp. |
| *Andrena miranda* | Hymenoptera | Andrenidae | And mir |
| *Pseudopanurgus parvus* | Hymenoptera | Andrenidae | Pse.par. |

Species composition of effect group cluster 4.

| **Taxonomic group** | **Order** | **Family** | **Abbreviation** |
| --- | --- | --- | --- |
| *Andrena cineraria* | Hymenoptera | Andrenidae | And.cin. |
| *Andrena scotica* | Hymenoptera | Andrenidae | And.sco. |
| *Chloromyia sp* | Diptera | Stratomyidae | Chl.sp. |
| *Melanostoma spp* | Diptera | Syrphidae | Melanost.sp |
| *Nomada sp* | Hymenoptera | Apidae | Nom.sp. |
| *Osmia sp* | Hymenoptera | Megachilidae | Osm.sp. |
| *Sphecodes sp* | Hymenoptera | Halictidae | Sph.sp. |
| *Syrphus ribesii/vitripennis* | Diptera | Syrphidae | Syr.rib |

Species composition of effect group cluster 5.

| **Taxonomic group** | **Order** | **Family** | **Abbreviation** |
| --- | --- | --- | --- |
| *Anthophora sp* | Hymenoptera | Apidae | Ant.sp. |
| *Apis mellifera* | Hymenoptera | Apidae | Api.mel. |
| *Athalia rosae* | Hymenoptera | Tenthredinidae | Ath.ros. |
| *Bombus borealis* | Hymenoptera | Apidae | Bom.bor. |
| *Bombus flavifrons* | Hymenoptera | Apidae | Bom.fla. |
| *Bombus frigidus* | Hymenoptera | Apidae | Bom.fri. |
| *Bombus hortorum* | Hymenoptera | Apidae | Bom.hor. |
| *Bombus hypnorum* | Hymenoptera | Apidae | Bom.hyp. |
| *Bombus impatiens* | Hymenoptera | Apidae | Bom.imp. |
| *Bombus jonellus* | Hymenoptera | Apidae | Bom.jon. |
| *Bombus lapidarius* | Hymenoptera | Apidae | Bom.lap. |
| *Bombus melanopygus* | Hymenoptera | Apidae | Bom.mel. |
| *Bombus nevadensis* | Hymenoptera | Apidae | Bom.nev. |
| *Bombus pascuorum* | Hymenoptera | Apidae | Bom.pas. |
| *Bombus pratorum* | Hymenoptera | Apidae | Bom.pra. |
| *Bombus Psithyrus sp* | Hymenoptera | Apidae | Bom.Psyth.sp. |
| *Bombus rufocinctus* | Hymenoptera | Apidae | Bom.ruf. |
| *Bombus soroeensis* | Hymenoptera | Apidae | Bom.sor. |
| *Bombus subterraneus* | Hymenoptera | Apidae | Bom.sub. |
| *Bombus sylvarum* | Hymenoptera | Apidae | Bom.syl. |
| *Bombus ternarius* | Hymenoptera | Apidae | Bom.ter. |
| *Bombus terr/luc* | Hymenoptera | Apidae | Bom.terr.luc |
| *Bombus terricola* | Hymenoptera | Apidae | Bom.terric. |
| *Bombus vagans* | Hymenoptera | Apidae | Bom.vag. |
| *Episyrphus balteatus* | Diptera | Syrphidae | Epi.bal. |
| *Eristalis spp* | Diptera | Syrphidae | Eri.sp. |
| *Helophilus spp* | Diptera | Syrphidae | Hel.sp. |
| *Pieridae mostly* | Lepidoptera | Pieridae | Pieridae |
| *Platycheirus sp* | Diptera | Syrphidae | Plat.sp. |
| *Polistes sp* | Hymenoptera | Vespidae | Pol.sp. |
| *Xylocopa sp* | Hymenoptera | Apidae | Xylocopa |

Species composition of effect group cluster 6. Note this group represents three species not assigned to any other group based on the au *p* values.

| **Taxonimc group** | **Order** | **Family** | **Abbreviation** |
| --- | --- | --- | --- |
| *Ceratina sp* | Hymenoptera | Apidae | Cer.jap. |
| *Hylaeus sp* | Hymenoptera | Colletidae | Hya.sp. |
| *Megachile sp* | Hymenoptera | Megachilidae | Meg.sp. |

**References**

1 Jauker, F. & Wolters, V. Hover flies are efficient pollinators of oilseed rape. *Oecologia* **156**, 819-823 (2008).

2 Jauker, F., Bondarenko, B., Becker, H. C. & Steffan-Dewenter, I. Pollination efficiency of wild bees and hoverflies provided to oilseed rape. *Agr. For. Entomol.* **14**, 81-87 (2012).

3 Steffan-Dewenter, I. Seed set of male-sterile and male-fertile oilseed rape (Brassica napus) in relation to pollinator density. *Apidologie* **34**, 227-235 (2003).

4 Garratt, M. P. D. *et al.* The identity of crop pollinators helps target conservation for improved ecosystem services. *Biol. Conserv.* **169**, 128-135 (2014).

5 Soroka, J. J., Goerzen, D. W., Falk, K. C. & Bett, K. E. Alfalfa leafcutting bee (Hymenoptera: Megachilidae) pollination of oilseed rape (Brassica napus L.) under isolation tents for hybrid seed production. *Can. J. Plant. Sci.* **81**, 199-204 (2001).

6 Lindström, S. A. M., Herbertsson, L., Rundlöf, M., Smith, H. G. & Bommarco, R. Large-scale pollination experiment demonstrates the importance of insect pollination in winter oilseed rape. *Oecologia* **180**, 759-769 (2016).

7 Bommarco, R., Marini, L. & Vaissiére, B. E. Insect pollination enhances seed yield, quality, and market value in oilseed rape. *Oecologia* **169**, 1025-1032 (2012).

8 Woodcock, B. A. *et al.* Spill-over of pest control and pollination services into arable crops. *Agric. Ecosyst. Environ.* **231**, 15-23 (2016).

9 Stanley, D., Gunning, D. & Stout, J. Pollinators and pollination of oilseed rape crops (Brassica napus L.) in Ireland: ecological and economic incentives for pollinator conservation. *J. Insect Conserv.*, 1-9 (2013).

10 Morandin, L. A. & Winston, M. L. Wild bee abundance and seed production in conventional, organic, and genetically modified canola. *Ecol. Appl.* **15**, 871-881 (2005).

11 Zou, Y. *et al.* Wild pollinators enhance oilseed rape yield in small-holder farming systems in China. *BMC Ecol.* **17**, 6 (2017).

12 Cook, R. D. *Residuals and Influence in Regression*. (Chapman & Hall, 1982).

13 Howlett, B. G. *et al.* Can insect body pollen counts be used to estimate pollen deposition on pak choi stigmas? *NZ Pl. Protection* **64**, 25-31 (2011).

14 Rader, R. *et al.* Alternative pollinator taxa are equally efficient but not as effective as the honeybee in a mass flowering crop. *J. Appl. Ecol.* **46**, 1080-1087 (2009).

15 Pollard, E. & Yates, T. J. *Monitoring Butterflies for Ecology and Conservation*. (Chapman and Hall, 1993).

16 Woodcock, B. A. *et al.* Crop flower visitation by honeybees, bumblebees and solitary bees: small scale behavioural differences linked to landscape scale responses. *Agric. Ecosyst. Environ.* **171**, 1-8 (2013).
