# Supplementary material for: Meta-analysis reveals that pollinator functional diversity and abundance enhance crop pollination and yield
Source: Nat Commun. 2019 Apr 1;10:1481. doi: 10.1038/s41467-019-09393-6 (PMC6443707; doi:10.1038/s41467-019-09393-6)
Supplement: Supplementary file 3 — Reporting Summary [file 41467_2019_9393_MOESM3_ESM.pdf]

nature research

Corresponding author(s): *Double-blind peer review submissions: write DBPR and your manuscript number here instead of author names*

Reporting Summary

Nature Research wishes to improve the reproducibility of the work that we publish. This form provides structure for consistency and transparency in reporting. For further information on Nature Research policies, see Authors & Referees and the Editorial Policy Checklist.

Statistical parameters

When statistical analyses are reported, confirm that the following items are present in the relevant location (e.g. figure legend, table legend, main text, or Methods section).

n/a	Confirmed	
☐	☒	The exact sample size (*n*) for each experimental group/condition, given as a discrete number and unit of measurement
☐	☒	An indication of whether measurements were taken from distinct samples or whether the same sample was measured repeatedly
☐	☒	The statistical test(s) used AND whether they are one- or two-sided<br>*Only common tests should be described solely by name; describe more complex techniques in the Methods section.*
☐	☒	A description of all covariates tested
☐	☒	A description of any assumptions or corrections, such as tests of normality and adjustment for multiple comparisons
☐	☒	A full description of the statistics including central tendency (e.g. means) or other basic estimates (e.g. regression coefficient) AND variation (e.g. standard deviation) or associated estimates of uncertainty (e.g. confidence intervals)
☐	☒	For null hypothesis testing, the test statistic (e.g. F, t, r) with confidence intervals, effect sizes, degrees of freedom and P value noted<br>*Give P values as exact values whenever suitable.*
☒	☐	For Bayesian analysis, information on the choice of priors and Markov chain Monte Carlo settings
☐	☒	For hierarchical and complex designs, identification of the appropriate level for tests and full reporting of outcomes
☐	☒	Estimates of effect sizes (e.g. Cohen's d, Pearson's r), indicating how they were calculated
☐	☒	Clearly defined error bars<br>*State explicitly what error bars represent (e.g. SD, SE, CI)*

Our web collection on statistics for biologists may be useful.

Software and code

Policy information about availability of computer code

Data collection	no software used
Data analysis	All analyses were undertaken in the open source R 3.5.0 statistical environment using the additional packages Metafor, MuMIn, Picante, FD, lme4.

For manuscripts utilizing custom algorithms or software that are central to the research but not yet described in published literature, software must be made available to editors/reviewers upon request. We strongly encourage code deposition in a community repository (e.g. GitHub). See the Nature Research guidelines for submitting code & software for further information.

Data

Policy information about availability of data

All manuscripts must include a data availability statement. This statement should provide the following information, where applicable:

- Accession codes, unique identifiers, or web links for publicly available datasets
- A list of figures that have associated raw data
- A description of any restrictions on data availability

Supplementary source data file (source-data.xlsx) contains raw and corrected abundance data and derived plot level community metrics used to determine

Pearson's correlations that were used in the meta-analysis. This file also contains all values presented in the figures. Supporting information contains all trait data (Supplementary Tables 2-3).

Field-specific reporting

Please select the best fit for your research. If you are not sure, read the appropriate sections before making your selection.

☐ Life sciences ☐ Behavioural & social sciences ☒ Ecological, evolutionary & environmental sciences

For a reference copy of the document with all sections, see nature.com/authors/policies/ReportingSummary-flat.pdf

Ecological, evolutionary & environmental sciences study design

All studies must disclose on these points even when the disclosure is negative.

Study description

We used a meta-analysis to test for correlations between measures of insect pollinator community structure (abundance, species richness, effect group richness, functional divergence, phylogenetic mean pairwise distance and CWM of body length, stigmal contact and hairiness index) and the yield of the crop oilseed rape under field and mesocosm conditions.

Research sample

The research sample data for use in the meta-analysis was derived using a web of science search that is fully described in the paper supplementary material in a Preferred Reporting Items for Systematic Reviews and Meta-Analyses (PRISMA) flow diagram. Each experiment was sub-divided into 'studies' that included observations undertaken on only a single variety within a single year. The experiments were based on two distinct methodologies, mesocosm experiments and field observations. In all subsequent analyses' studies from these two distinct methodologies were analysed separately. For mesocosm experiments, individual mesocosms were treated as replicates. For field experiments, a plot observed for a single year was treated as a replicate, and data on pollinator communities assessed at shorter time scales were summed. Where field experiments included zero abundance controls in the form of exclusion cages these were treated as separate data points equivalent to those used for the mesocosm studies. From these studies Pearsons correlations between measures of pollinator community structure and yield of oilseed rape would be derived and used as the samples within the meta-analysis presented in the paper. Published studies where data was obtained for inclusion in the meta-analysis are Bommarco, R., Marini, L. & Vaissiére, B. E. Insect pollination enhances seed yield, quality, and market value in oilseed rape. Oecologia 169, 1025-1032 (2012); Garratt, M. P. D. et al. The identity of crop pollinators helps target conservation for improved ecosystem services. Biol. Conserv. 169, 128-135 (2014); Jauker, F. & Wolters, V. Hover flies are efficient pollinators of oilseed rape. Oecologia 156, 819-823 (2008); Jauker, F., Bondarenko, B., Becker, H. C. & Steffan-Dewenter, I. Pollination efficiency of wild bees and hoverflies provided to oilseed rape. Agric. For. Entomol. 14, 81-87 (2012); Lindström, S. A. M., Herbertsson, L., Rundlöf, M., Smith, H. G. & Bommarco, R. Large-scale pollination experiment demonstrates the importance of insect pollination in winter oilseed rape. Oecologia 180, 759-769 (2016); Morandin, L. A. & Winston, M. L. Wild bee abundance and seed production in conventional, organic, and genetically modified canola. Ecol. Appli. 15, 871-881 (2005); Soroka, J. J., Goerzen, D. W., Falk, K. C. & Bett, K. E. Alfalfa leafcutting bee (Hymenoptera: Megachilidae) pollination of oilseed rape (Brassica napus L.) under isolation tents for hybrid seed production. Can. J. Plant. Sci. 81, 199-204 (2001); Stanley, D., Gunning, D. & Stout, J. Pollinators and pollination of oilseed rape crops (Brassica napus L.) in Ireland: ecological and economic incentives for pollinator conservation. J. Insect Conserv., 1-9 (2013); Steffan-Dewenter, I. Seed set of male-sterile and male-fertile oilseed rape (Brassica napus) in relation to pollinator density. Apidologie 34, 227-235 (2003); Woodcock, B. A. et al. Crop flower visitation by honeybees, bumblebees and solitary bees: small scale behavioural differences linked to landscape scale responses. Agric. Ecosyst. Environ. 171, 1-8 (2013); Woodcock, B. A. et al. Spill-over of pest control and pollination services into arable crops. Agric. Ecosyst. Environ. 231, 15-23 (2016); Zou, Y. et al. Wild pollinators enhance oilseed rape yield in small-holder farming systems in China. BMC Ecol. 17, 6 (2017).

Sampling strategy

No sample size calculation was undertaken. for the meta-analysis. All available data from published sources was used in the meta-analysis.

Data collection

The research sample data for use in the meta-analysis was derived using a web of science search that is fully described in the paper supplementary material in a Preferred Reporting Items for Systematic Reviews and Meta-Analyses (PRISMA) flow diagram. In summary, a Web of Science search under the criteria 'Oilseed rape' OR 'Canola' OR 'Rapeseed' OR 'Brassica napus' AND 'Pollination/ Pollinator(s)' AND 'Yield' was undertaken. This was complimented by additional experiments sought from other sources, including published and unpublished studies (see Supplementary Methods 4 for methodologies used to derive data in these unpublished studies where included in the meta-analyses). This produced a total of 145 experiments. These were checked on the basis of eligibility criteria: 1) studies contained a direct measure of oilseed rape yield recorded and associated within individual experimental units; 2) insect pollinator communities were quantified to species or similar high-resolution taxonomic units (see below for details); 3) studies contained at least 4 experimental units allowing a variance to be derived. This derived a sub-set of 18 experiments which were then subdivided on the basis of the varietal type of oilseed rape grown. Raw yield and species level data were sought direct form original authors of the studies included in the meta-analysis with these being processed by the lead author paper author BAW. This produced a finalised data set of 23 studies for inclusion in two separate meta analyses, one for mesocosm based studies (7 sutures) and one for field based studies (16 studies). As a meta-analysis individual studies represent the sample units, with sample size used to determine a measure of variance. Raw data on species came in a range of taxonomic resolutions depending on the study. Individual field studies identified Hymenoptera (e.g. Apoidea, Vespidae and Tenthredinidae), Diptera (principally Syrphidae) and Lepidoptera to a species, genus or functional level (Supplementary Tables S1). Genus classifications were used when reliable identification to species was not consistent across studies (e.g. Lasioglossum, Hylaeus, Megachile, Halictus and Osmia). Functional types were used for taxonomically complex groups. For example, the predominately Calypterate flies, while composed principally of Delia sp. (Anthomyiidae), included other families such as Calliphoridae and Muscidae. Similarly, as 97.5% (total N=1,139) of all butterfly individuals were Pieris spp. (Pieridae) other butterfly species were combined into a single functional group. For mesocosm studies, exact species compositions and abundances were always known. However, for field-based experiments abundance was quantified at the scale of a field using either pan traps or transect / quadrat-based observations. Within each study abundance values

	were transformed to have a common standard deviation of 1, although were not corrected to a mean of zero so that zero abundance plots remained zero. For each experimental replicate (either field or mesocosm) a summed abundance and species richness was derived.
Timing and spatial scale	The meta analysis was based on a Web of Science search run from WOS search from 1980 – March 2018 (the point as which data processing and subsequent analysis was initiated).
Data exclusions	Within individual studies included in the meta-analysis species community data was subject to data exclusion. Species with ≤ 5 individuals across all data sets were excluded to minimise the effect of potentially transient species moving though fields but not foraging directly on the crop. Although coleoptera and parasitic Hymenoptera were recorded for some studies the variable taxonomic resolution applied meant that these were excluded from the analysis. During the meta-analyses studies were excluded where model diagnostic plots identified large influence. The number of studies excluded is indicated both in the main results and in greater detail in the supplementary material.
Reproducibility	As a meta-analysis we were using data sets obtained from previously published studies investigating the link between pollinator communities and oilseed rape yield. These studies have undergone peer review. In no case were individual studies repeated under the same experimental conditions to assess the level of repeatability of the results.
Randomization	Individual studies whose Pearsons correlation between pollinator community structure and oilseed rape yield were used as raw data in the meta-analysis were allocated to groups on the basis of: 1) the yield metric used in the study; 2) the breeding type of the oilseed rape plant; 3) the sterility of the oilseed rape plant. These groupings were included in the meta-analyses as moderating factors to account for this between study heterogeneity.
Blinding	No blinding was used during data acquisition.

Did the study involve field work? ☐ Yes ☒ No

Reporting for specific materials, systems and methods

Materials & experimental systems

n/a	Involved in the study
☒	☐ Unique biological materials
☒	☐ Antibodies
☒	☐ Eukaryotic cell lines
☒	☐ Palaeontology
☒	☐ Animals and other organisms
☒	☐ Human research participants

Methods

n/a	Involved in the study
☒	☐ ChIP-seq
☒	☐ Flow cytometry
☒	☐ MRI-based neuroimaging
