# Supplementary material for: Meta-analysis reveals that pollinator functional diversity and abundance enhance crop pollination and yield
Source: Nat Commun. 2019 Apr 1;10:1481. doi: 10.1038/s41467-019-09393-6 (PMC6443707; doi:10.1038/s41467-019-09393-6)
Supplement: Supplementary file 4 — Description of Additional Supplementary Files [file 41467_2019_9393_MOESM4_ESM.pdf]

Description of Additional Supplementary Files

File Name: Supplementary Data 1
Description: To identify combinations of traits that predict oilseed rape yield (corrected to one SD) we used a general linear mixed model with all combinations of the pollinator effect trait given as community weighted means (CWM). This table shows the 53 models (representing 0.3 % of the 16384 tested models) that fall within 2 AIC of the single best fit model. These have equivalent explanatory power for the data. For each model we present log likelihood values (LogLik), AIC, ΔAIC (difference in AIC value from best fit model) and Akaike weights (wi) which describe the probability that a given model would be selected as the best fitting model if the data were recollected. For each of the 14 considered CWM effects traits (note 4 effects traits were excluded from the analysis, see Methods section) we derive: 1) percentage of the ΔAIC≤2 models that contain the CWM effect trait as an explanatory factor; 2) a variable importance (VI) parameter which sums wi values over all models containing that explanatory CWM trait value and ranges between 0 and 1; and 3) an averaged parameter estimate for the correlation with between CWM trait value and yield weighted by the wi values. Note for each model we present individual parameter estimates of the correlation where it was present in that model.

File Name: Supplementary Data 2
Description: Taxonomic associations of species (used for the Phylogenetic Mean Pairwise Distance) and derived morphological and behavioural effect traits for each species. Traits are defined in Table 2 in the main paper. These traits are determined from direct behavioural observation of bees on oilseed rape flowers (average time on flower when foraging and probabilities of nectar foraging, pollen foraging, and dry pollen on the body) and information on morphology from taxonomic and other literature. Where data on individual species was not available (e.g. behavioural observations) generic or higher order classification means were used.

File Name: Supplementary Data 3
Description: This data file details values used in the derivation of the body hairiness index. For each species body parts that come into contact with oilseed stigmas (head, thorax sternum, abdomen underside, and femora, tibiae and meta-tarsus (legs assessed separately) were scored as: 0) coarse setae or extremely short hairs; 1) short (c. basal tibiae 1 diameter) but dense hairs (>50 mm2); 2) long (>basal tibiae 1 diameter) dense (>50 mm2) hairs. This score was summed and given as a percentage of the maximum score of 24 to provide an index that ranged from 0 (not hairy) to 1 (very hairy).

File Name: Supplementary Data 4
Description: Data file containing the raw abundances of insect pollinators from the 17 studies used in the meta-analyses. For subsequent analyses all raw abundances were standardised within a study to have a standard deviation of zero.
